# Challenges in the Conduct of Research: Chemotherapy-Induced Peripheral Neuropathy

**Published:** 2013-09-01

**Authors:** Constance Visovsky

**Affiliations:** From University of South Florida College of Nursing, Tampa, Florida

The treatment of chemotherapy-induced peripheral neuropathy (CIPN) and associated neuropathic pain continues to be of significant concern to patients and clinicians. Peripheral neuropathy is a condition resulting from damage to peripheral nerves (Stubblefield et al., 2009). Chemotherapy-induced peripheral neuropathy is considered a dose-limiting side effect of known neurotoxic cancer agents (Pachman, Barton, Watson, & Loprinzi, 2011). Sensory symptoms of CIPN include numbness, tingling, and increased sensitivity to touch and cold temperatures. Neuropathic pain is one aspect of the CIPN experience, affecting approximately 20% to 40% of patients treated for cancer with known neurotoxic agents (Kautio, Haanpaa, Kautiainen, Kalso, & Saarto, 2011; Loprinzi et al., 2011; Smith, Cohen, Pett, & Beck, 2010). Neuropathic pain is often described as burning, lancinating, freezing, or electric shock-like (Stubblefield et al., 2009). While CIPN remains a pervasive problem, there are challenges to the conduct of research in the identification and treatment of CIPN.

## Study Design

Symptom management studies require careful consideration of study design. Considerations of sample inclusion, exclusion criteria, sample size, and outcome and control variables that impact the analytic plan also must be noted. The use of a crossover design reduces the number of potential confounding variables by having each participant serve as his or her own "control." This also decreases the sample size needed to study to phenomenon of interest, so it is considered efficient and cost-effective. However, one must also consider that the order in which each participant gets the intervention may affect the study outcome.

The design of a study is an important factor that can certainly impact the study outcome. Therefore, factors that can impact results of intervention studies for CIPN must be carefully considered. In many studies of pharmacologic and nonpharmacologic therapies for the prevention and treatment of CIPN, the sample often includes a mix of cancer diagnoses, time since diagnosis, and treatments received. The researcher must consider how each of these variables will be handled in the inclusion/exclusion criteria of the study and how they may be controlled using advanced statistical techniques. For example, in the duloxetine study (Smith et al., 2013) reviewed on page 361 of this issue by Rita Wickham, the study protocol permitted inclusion of patients with any cancer diagnosis or stage experiencing neuropathic pain for 3 or more months following the completion of chemotherapy. The lack of homogeneous sampling can potentially impact the findings of CIPN therapies, which can appear nonefficacious or unclear as to which therapies work best for a specific neurotoxic agent.

In the duloxetine study (Smith et al., 2013), the initial inclusion criteria were expanded from patients receiving oxaliplain and paclitaxel to also include patients treated with single-agent docetaxel, nanoparticle albumin-bound paclitaxel or cisplatin. The large sample size and statistical technique for stratification of the sample allowed for consideration of the chemotherapy drug received and are strengths of this study. In studies of CIPN, the interaction of comorbidity and cancer treatment is a crucial factor in study design. In the duloxetine study, consideration of important comorbid illnesses that can impact study results was well specified and taken into account in balancing the intervention groups and in the analysis. In addition, the researchers controlled for use of selected analgesics and excluded the use of other medications that could influence serotonin levels. The consideration of key control variables underscores the importance of consulting with a statistician during the initial design phase of the study.

## Measurement Issues

One of the most significant challenges to the study of CIPN is the lack of a gold standard definition and measure. In the duloxetine study by Smith et al., CIPN was diagnosed based upon symptomatology, loss of deep-tendon reflexes, or the presence of symmetric stocking-glove numbness or paresthesias that began after the start of neurotoxic chemotherapy. The symptom history that contributed to the determination of CIPN in the duloxetine study in addition to the presence of symmetric numbness/paresthesia of the extremities is not clear.

The Common Terminology Criteria for Adverse Events (CTCAE) is the most commonly used instrument to measure CIPN in research studies and clinical trials. Despite the widespread use of the CTCAE, significant issues related to its use remain. There is a lack of standardization and consensus regarding training required for scoring an instrument that contains both subjective and objective assessment, as well as inconsistent interpretation, resulting in substantial interobserver disagreement. The CTCAE lacks comprehensive aspects for objective, testing with the exception of assessment of deep-tendon reflexes (CTCAE, v.4.0). There is no assessment of touch sensation, pinprick, vibration, or proprioception included. The terminology of the instrument is rather vague, with terms such as "moderate symptoms" without explicating specific symptoms that each examiner may inquire about. The resulting variation in use and interpretation of the CTCAE may result in discrepancies in identification of functional deficits that pose difficulty in instrumental activities of daily living for patients.

The timing of interventional measures and the length of follow-up that takes into consideration the "coasting effect" of certain neurotoxic agents, where CIPN may actually worsen following the discontinuation of chemotherapy before improvements are seen, are both confounding factors. In light of these deficiencies, investigators should comtemplate including a multisymptom instrument in addition to the CTCAE in the assessment and grading of CIPN (Visovsky, Berger, Kosloski, & Kercher, 2008).

## Data Analysis

Lastly, the plan for analysis of study data is an important concern in research where symptoms such as CIPN are the outcome of interest. When chemotherapy is treated in analysis by dummy coding (chemotherapy/no chemotherapy), this approach fails to capture the variance of the timing, duration, and severity of CIPN, which can vary widely in differing cancer populations and treatment regimens. The duloxetine study took several analytic approaches, including change in average pain score from start of initial treatment over a 5-week period, exploratory responder analyses, examination of secondary quality-of-life endpoints and three models of analysis of covariance stratified by chemotherapy agent, risk of neuropathy, and baseline measure of the desired endpoint (Smith et al., 2013). This thorough approach to the data analysis took into account all possible influences on the study endpoint of pain and also considered important covariates and other potential influences, such as analgesic use. The interference of pain on physical function and quality of life was also presented in the analysis.

Missing data are problematic in any longitudinal study, especially when compounded by subject attrition. Careful consideration needs to be taken in consultation with the study statistician with regard to the data imputation technique selected to avoid bias in the result. In the duloxetine study, there was a similar adverse event rate in both groups; however, the group receiving duloxetine experienced a greater dropout rate. The study analysis report noted that several multiple imputation models were used to evaluate the pattern and influences of missing data.

## Conclusion

The conduct of research in CIPN can be challenging. Yet there is a tremendous need for additional research into the prevention and treatment of CIPN to improve quality of life and prevent or ameliorate physical disability. Investigations for the prevention or treatment of CIPN require consultation with a statistician who can assist in determining the sample size and power, consider the influence of attrition and missing data and appropriate measures for the study endpoints. One of the most important considerations in evaluating patients with CIPN is to determine how symptoms of functional deficits associated with peripheral neuropathy interfere with their activities of daily living, as these factors influence decisions to delay treatment, reduce the treatment dose, or cease treatment altogether (Hausheer, Schilsky, Bain, Berghorn, & Lieberman, 2006). Advanced practitioners should be able to critique symptom management research with consideration of the issues of study design, measurement, and analysis to evaluate whether the results are indeed valid and can be considered for application to practice.
